# Protocol for a cervical screening implementation trial comparing two approaches for delivering HPV self-collection in low-resource settings in India: a type 3 hybrid cluster randomised controlled trial (SHE-CAN)

**DOI:** 10.1136/bmjopen-2025-101599

**Published:** 2025-12-29

**Authors:** Anu Mary Oommen, Maleeha Ashfaq, Mary Vanlalhruaii Tonsing, Anne George Cherian, Pravin Singarayar, Vidhya Viswanathan, Venugopal Muniswamy, David Hawkes, Priya Abraham, Ruby Angeline Pricilla, Ravikumar Manoharan, Eric Zomawia, Brian Oldenburg, Marion Saville, K Krishnaraj, Selvavinayagam T S, Partha Basu, Julia ML Brotherton, on behalf of the SHE-CAN collaborators, Abraham Peedicayil

**Affiliations:** 1 Centre for Health Policy, Melbourne School of Population and Global Health, The University of Melbourne, Melbourne, Victoria, Australia; 2 Christian Medical College Vellore, Vellore, Tamil Nadu, India; 3 Population Based Cancer Registry, Dept of Pathology, Civil Hospital Aizawl, Aizawl, Mizoram, India; 4 Tribal Health Initiative, Sittilingi, Tamil Nadu, India; 5 Directorate of Public Health and Preventive Medicine, Government of Tamil Nadu, Chennai, Tamil Nadu, India; 6 Australian Centre for the Prevention of Cervical Cancer, Melbourne, Victoria, Australia; 7 Baker Heart and Diabetes Institute, Melbourne, Victoria, Australia; 8 International Agency for Research on Cancer, Lyon, France

**Keywords:** Human Papillomavirus Viruses, Implementation Science, PUBLIC HEALTH

## Abstract

**Background:**

Although multiple studies have offered self-collection for human papillomavirus (HPV)-based cervical screening in community settings, there are no randomised controlled trials (RCTs) that have compared implementation outcomes of programme approaches for self-collection. This trial will compare two such approaches in low-resource settings in the states of Tamil Nadu and Mizoram, India.

**Methods:**

A cluster RCT will be conducted over a year, offering self-collection to 3000 women aged 30–49 from 28 clusters (average size 101) in selected districts. Clusters in tribal, rural and urban low-income settings will be randomised to two arms. The intervention arm, co-designed with multiple stakeholders, will involve campaigns to offer self-collection in the community. The comparison arm will be offered self-collection at the nearest health facilities.

HPV-based cervical screening will be performed at central laboratories using clinically validated screening assays that can identify the highest risk carcinogenic HPV types (Group 1a–c - HPV16/18/31/33/45/52/58, ±35). Ablative treatment will be based on positivity with this extended genotyping triage, while those with any of the lower carcinogenic HPV types (Group 1d - 39, 51, 56, 59, ±35, Groups 2a/b - 66, 68) will undergo further assessment with visual inspection with acetic acid. Outcomes will be evaluated quantitatively and qualitatively using RE-AIM and the Theoretical Framework of Acceptability.

**Analysis:**

The primary outcome will be percentage of women well-managed (screened and appropriately treated) in both arms, with secondary outcomes including proportion screened, proportion treated, acceptability (willingness to screen, rescreen, and/or recommend to others) to women, community and healthcare providers, adoption (by providers), implementation fidelity, costs, sustainability assessment and systematically identified implementation barriers and facilitators. The reach, effectiveness and acceptability of community-based self-collection and the use of extended genotyping for triage in resource-constrained, hard-to-reach populations will be assessed, with lessons that can inform future statewide and national programmes.

**Ethics and dissemination:**

Ethics approval has been obtained from the Institutional Review Board (IRB) and Ethics Committee of the Christian Medical College Vellore, Tamil Nadu, India (IRB Min. No 14314; INTERVEN), the Alfred Hospital Ethics Committee (HREC Ref 80134, Local Reference: project 601/21), Melbourne, Australia, the IARC Ethics Committee (IEC 21-32), Lyon, France, the Salem Polyclinic Institutional Ethics Committee (SPCIEC/2022/June/01/02), Tamil Nadu, India and the Institutional Ethics Committee, Civil Hospital, Aizawl, Mizoram, India (No.B.12018/1/13-CHA(A)/IEC/115). The study is also approved by the State Scientific Advisory Committee, Directorate of Public Health and Preventive Medicine, Chennai, Tamil Nadu (R. No. 011575/HEB/A2/2023). The Alfred Hospital Approval, as an authorised Australian ethics committee for national mutual recognition, is recognised and registered with the University of Melbourne Human Research Ethics Committee (2024-25255-57650-1). Written informed consent will be obtained from participants. The results of the trial will be disseminated through a peer-reviewed medical journal, and also through workshops, reports and conferences.

**Trial registration number:**

The trial has been registered with the Clinical Trials Registry - India: CTRI/2022/04/042327.

**STRENGTHS AND LIMITATIONS OF THE STUDY**

- First cluster randomised controlled trial from India comparing two implementation approaches for offering self-collection.
- Comparison of a co-designed community outreach-based self-collection approach against facility-based self-collection.
- Generalisability is limited to similar settings and populations in India.

## Background

 In the context of the WHO’s call for the elimination of cervical cancer as a public health problem, Indian policymakers are faced with an urgent need for evidence on how to implement cervical screening to achieve population-level coverage effectively.^1^ One of the scale-up targets for the global strategy is to achieve, by 2030, a 70% coverage of twice in a lifetime cervical screening, using nucleic acid testing for human papillomavirus (HPV) (or test of equivalent precision if one becomes available) to prevent cervical cancer, a recommendation that applies across settings. While India’s current policy solution of Visual Inspection of the cervix with Acetic acid (VIA)^2^ is primarily based on affordability, the coverage achieved is low (1.9% ever screened)^3^ due to multiple provider and recipient-related barriers.^4^ It is of critical importance that screening is scaled up in India, which reported almost 80 000 deaths due to cervical cancer in 2022.^5^

HPV-based cervical screening every 5 years is more accurate and cost-effective than 3 yearly VIA screening and decreases both incidence and mortality due to cervical cancer.^6 7^ An additional and significant benefit of HPV-based screening is that vaginal samples collected via self-collection, hereafter referred to as self-collection, can detect HPV at levels indicative of precancer of the cervix (cervical intraepithelial neoplasia grade 2 or above—CIN2+) as accurately as cervical samples collected using a speculum by a clinician.^8^ Most women screened will not have HPV detected, meaning that they can avoid a speculum examination altogether, and resources can be focused on those women in whom HPV is detected and who are at an increased risk of having CIN2+, and of developing cervical cancer. Self-collection is highly acceptable across settings internationally and helps overcome some of the major barriers to cervical screening using cytology or VIA, including stigma, shame, embarrassment, past trauma, fear and shortage of time, space, and healthcare workers to undertake speculum-based screening.^9^,^13^

Self-collection has been offered for cervical screening in interventional non-randomised studies across various states in India,^14^ but there has been no randomised controlled implementation research trial comparing possible options for delivering self-collection programmes. Implementation approaches include home-based testing, health centre-based testing and health promotion and education approaches such as one-on-one counselling, pamphlets and group education.^915^,^18^ India is both culturally and geographically diverse, requiring an HPV-based screening approach that is contextually appropriate in its implementation. Vulnerable groups such as tribal populations have shown a higher prevalence of HPV infection and also face significant challenges to screening and follow-up.^16 19^ Evidence-based contextualised approaches to delivering programmes are required for such populations in each state, given issues with both challenges to health systems, as well as cultural and socioeconomic inequities that lead to lower utilisation of health services in general.^20^

The formative phase of the SHE-CAN (Self-collected HPV evaluation for the prevention of cervical cancer) study used mixed methods research based on the INSPIRE (Integrative Systems Praxis for Implementation Research) methodology.^21^ This phase was conducted to understand the current implementation of VIA screening and to assess readiness of communities and the health system to adopt self-collection in two states of India: Tamil Nadu in Southern India and Mizoram in Northeastern India.^22^ These states have higher rates of cervical cancer incidence and mortality compared with most other Indian states, with greatly differing health systems and cultures.^23^

Following the completion of the formative phase and stakeholder workshops to co-design the trial arms, we now outline the protocol for a pragmatic cluster RCT to compare two different programme approaches to offering self-collection for HPV-based screening for vulnerable populations in Tamil Nadu and Mizoram.

We aim to compare implementation outcomes for two possible programme models: (1) The co-designed community-outreach approach for self-collection and (2) A facility-based self-collection approach, similar to the current model in place for VIA screening. The primary objective is to compare the percentage of well-managed women in each arm (screened and appropriately managed based on test outcomes). Secondary outcomes include differences in proportions of target population invited and screened during the trial period, compliance to further assessment or treatment, acceptability of self-sampling and determinants of successful implementation. The screening coverage using self-collection will also be compared with previously reported coverage of the VIA screening programme in both states.

## Methods

### Study settings

The study setting will include Vellore, Tiruvannamalai and Dharmapuri districts in Tamil Nadu, and Mamit district in Mizoram. The majority of the study population in each district belongs to rural communities and low socioeconomic strata. Populations selected in Tiruvannamalai, Dharmapuri and Mamit are designated as Scheduled Tribes by the Indian government. These populations rate lower on social and health indicators compared with the rest of the population^24^ and form around 8.6% of the country’s population.^25^ Two not-for-profit study organisations (Christian Medical College Vellore and the Tribal Health Initiative) offer primary and secondary care services in such tribal areas in Tamil Nadu, while healthcare in Mamit district in Mizoram is provided by the state government.^22^ The SHE-CAN study settings represent similar physically and culturally hard-to-access populations present across the country that are rarely included in cervical screening trials. The state’s public healthcare facilities will be active partners with the two study organisations in Tamil Nadu, while in Mizoram, the study will be conducted by the Mizoram government’s Population Based Cancer Registry.

Table 1 provides details of study locations, populations and the number of health facilities available, such as Health Sub Centres (HSCs), Primary Health Centres (PHCs), Community Health Centres and higher-level facilities.

**Table 1:** Description of study settings for the implementation trial

District, state, type of setting,	Literacy rate	Population size of study areas	Estimated women 30–49 years, clusters available in study areas	Government health facilities	Private* study organisation’s health facilities
Mamit, Mizoram, tribal	85% (census 2011)	101 916 (2024 local census)	11 559 in 91 villages	39 HSCs, 8 PHCs, 1 CHC, 1 district hospital	No private health facility involvement
Vellore, Tamil Nadu, rural	79% (2024)	88 235 (2023 local census)	14 298 in 65 villages	15 HSCs, 3 PHCs, 1 CHC, 1 medical college	Rural 140-bed secondary hospital
Vellore, Tamil Nadu, urban poor	79% (2024)	15 202 (2024 local census)	2006 in 17 urban localities	14 HSCs, 3 Urban PHCs, 1 sub-district hospital	Urban 46-bed secondary hospital, 2500-bed tertiary hospital
Tiruvannamalai, Tamil Nadu, tribal Jawadhi hills	(50%) (2023)	51 400 (2023 local census, Malayali tribes)	7817 in 230 tribal hamlets (Malayali tribes)	10 HSCs, 1 PHC, 1 CHC	Tribal outpatient clinic with monthly gynaecology clinic
Dharmapuri, Tamil Nadu. Tribal Sittilingi valley	66% (census 2011)	12 000 (2019 local census)	1241 in 23 tribal hamlets (21 Malayali hamlets, 2 Lambadi hamlets)	2 PHCs, 4 HSCs	Tribal 35-bed secondary hospital

* Not-for-profit study organisations: Christian Medical College, Vellore, Tribal Health Initiative ’s tribal hospital

CHC, Community Health Centre; HSC, Health Sub Centre; PHCs, Primary Health Centres.

### Study design

We chose a two-arm parallel group cluster randomisation design to minimise the effect of contamination as cancer screening behaviour is related to peer opinions in Indian communities.^26^ We used appropriate statistical methods (described later) to calculate the sample size required to detect meaningful differences. Since community programmes may need to invite all eligible women living in geographically clustered communities to participate, we may recruit slightly larger numbers than required to achieve the estimated sample size.

The trial, which is a hybrid type 3 implementation-effectiveness study,^27^ will compare the effectiveness of a community-based self-collection approach (intervention arm) to facility-based self-collection (control) across rural, urban poor and tribal areas in the two selected states. We will use the RE-AIM framework^28^ to evaluate both of the arms for implementation research outcomes. The protocol has been prepared based on the Standard Protocol Items: Recommendations for Interventional Trials guidelines (online supplemental additional file 1).^29^

### Eligibility criteria

#### Participants

The age group targeted for HPV-based screening will be 30–49 years, in line with the WHO guidelines,^30^ as the possibility of detecting precancers is highest in this age group. All women in the selected clusters, aged 30–49 years, will be identified through a house-to-house enumeration survey to be conducted by field workers. Those with a previous history of cervical cancer, hysterectomy or treatment for precancer in the previous 12 months and those who have undergone HPV testing in the previous 5 years will be excluded. Currently, pregnant women and those within 12 weeks of delivery or abortion will also be excluded, as per current Indian screening guidelines.^31^ Those with symptoms of intermenstrual, post-menopausal or post-coital bleeding will be referred for a pelvic examination and appropriate clinical management as indicated, before offering self-collection.

#### Cluster selection

**Figure 1:**
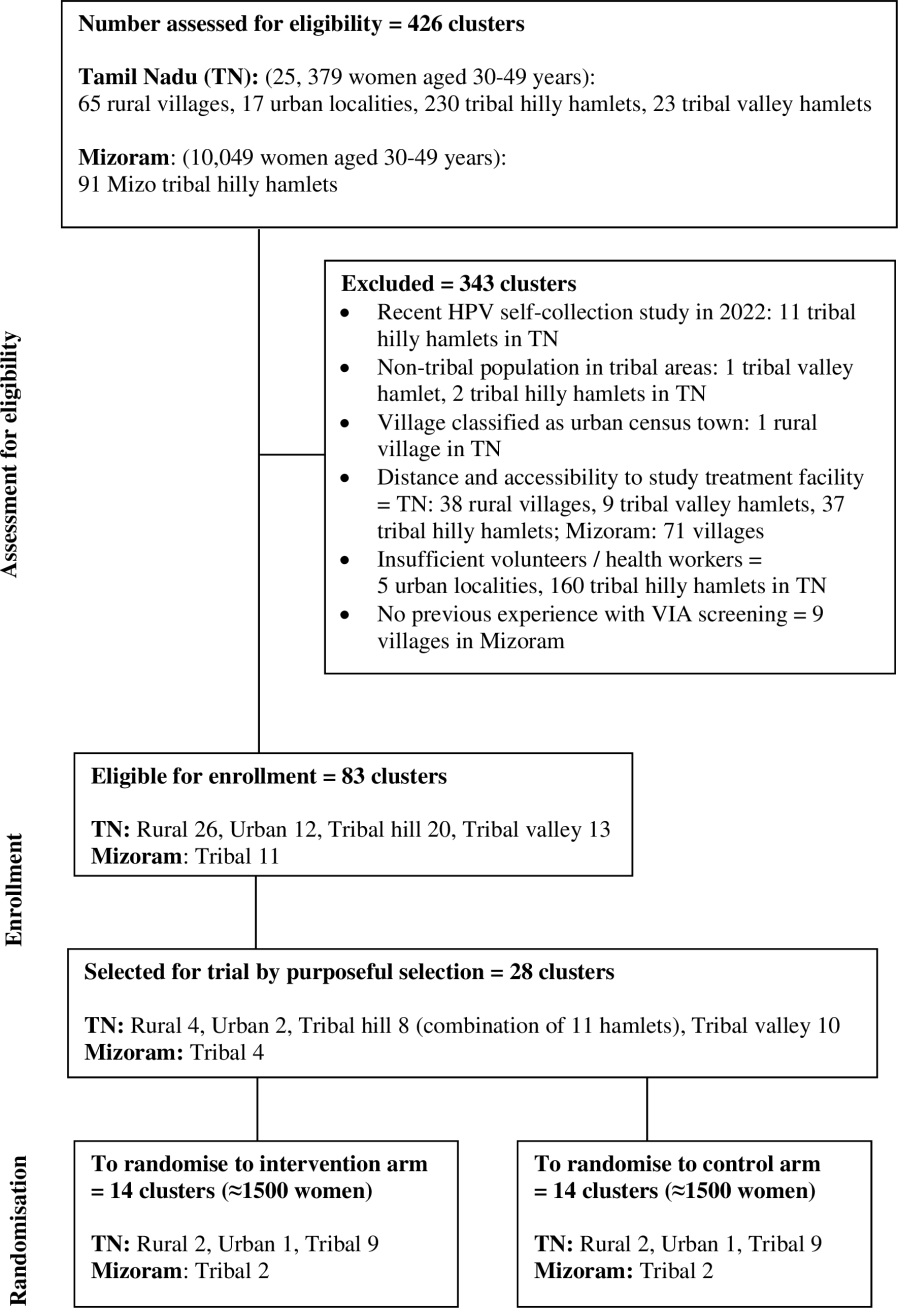
Flow diagram showing selection of clusters in study areas. HPV, human papillomavirus.

Villages, urban localities or tribal hamlets within the defined geographical areas were considered as clusters and the sample size estimation (described later) found that a total of 28 clusters needed to be randomised. A total of 426 clusters were assessed for eligibility, of which 343 were excluded, either because implementing the study would not be logistically feasible in those clusters or screening studies had already been implemented in those areas (figure 1). Selection of the final 28 clusters was done by the study team and local communities using purposive selection from the 83 clusters considered eligible for randomisation based on further considerations of feasibility such as availability of adequate staff, road access in tribal areas and geographical scattering of the population.

In rural Tamil Nadu, the size of the village was also considered while selecting clusters as there was a wide variation in cluster (village) size, as inclusion of very large villages would not be realistic for Community Health Workers (CHWs) to offer screening within the study duration of 12 months.

### Intervention arms

#### Selection of intervention

The interventions were selected through a formative phase,^22^ which included inputs from a variety of stakeholders following the steps of the INSPIRE implementation framework.^21^ The methods included a systematic review of previous HPV studies in India, desk review of the current screening programme, capacity assessment of health facilities and qualitative studies with communities and health providers. Information triangulated from the above exercise was shared with stakeholders in a co-design workshop as described below.

#### Co-design workshop to finalise interventions

The trial protocol was finalised following co-design workshops in both states, in 2024, involving community representatives, cancer survivors and CHWs along with public health nurses, public health physicians, gynaecologists, oncologists, virologists, researchers, programme managers and policy makers.

Stakeholders in all settings rejected the idea of same day screen and treat (using a point-of-care HPV test to give same day results) as this could be too overwhelming for women, pose difficulties in maintaining confidentiality of results and require a large number of women to wait for their test results on the same day in outreach clinics. Stakeholders emphasised the critical role of in-community group education and including males in community sessions, to facilitate community level acceptance and understanding. They also preferred a triage step for HPV positive women to reduce the potential for overtreatment.

### Description of co-designed interventions

**Figure 2:**
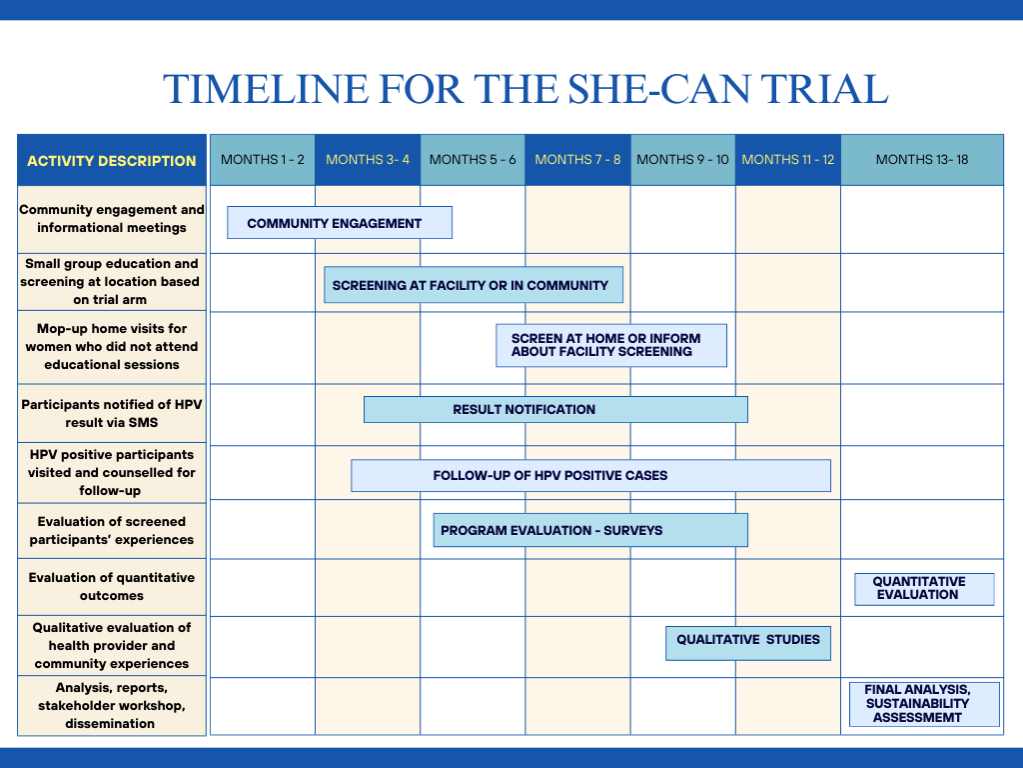
Timeline for the self-collected HPV evaluation for the prevention of cervical cancer trial. HPV, human papillomavirus; SHE-CAN, self-collected HPV evaluation for the prevention of cervical cancer.

Figure 2 depicts the timeline of activities for the implementation trial.

#### Arm one: community-based screening arm

Arm one will use a community-based outreach approach to offer self-collection in the community. An initial community meeting will be held in each community after engaging with local community leaders and obtaining their endorsement. Eligible women, older women and men will be invited to provide information and education about cervical screening, with a medical officer/nurse present to reassure women and their families about the effectiveness and safety of the self-collected HPV test. This large group meeting will be followed by smaller group sessions with eligible women, organised by CHWs/volunteers in locations such as Anganwadi (daycare) centres, schools, government buildings etc. CHWs will include both the study organisations’ health workers, local volunteers and the state government’s HSC level staff. These small group sessions will provide women with information on self-collection, with an offer for self-collection at the same locations. Women will also be given an option to self-collect samples in the privacy of their homes and bring back the samples to be handed over to the CHWs.

#### Arm two: facility-based screening (comparator arm)

Arm two will be similar to the community-based intervention arm, except for the location of screening. Initial community meetings will be followed by small group sessions for eligible women, similar to the intervention arm. However, in Arm two, women will only be informed about the availability of free self-collection at the nearest health facility, with no immediate kit distribution. The facilities where self-collection will be made available are either subcentres or PHCs, depending on proximity to study sites and usual site of VIA screening in that setting. This programme approach is similar to the current method of screening in these states, where Women Health Volunteers or ASHA workers (tribal) in Tamil Nadu and Health and Wellness Officers in Mizoram refer women for VIA screening to primary care facilities. Women not willing to self-collect the samples will have the option of the sample being taken at the facility by a trained nurse/health worker.

### Components common to both arms

#### Health education

Health education will primarily be through group sessions which will be held in both arms over the initial 6 months. Those who do not attend these group sessions will be approached later through door-to-door visits. During this mop-up phase, women in the community arm will be offered home-based screening, while in the facility arm, information will be given regarding availability of self-collection screening at the nearest health facility.

Educational content is being developed or adapted from existing sources. For Mizoram, based on feedback from local stakeholders, existing video content that has been used elsewhere has been identified as appropriate for the Mizo culture. https://www.youtube.com/watch?reload=9&v=CRNI-vyLjIw However, for Tamil Nadu, there was a need to develop new culturally appropriate content. As part of the formative phase of the trial, tribal female volunteers were invited to co-design and test a storyline, after receiving training on key messaging for HPV-based self-collection and cervical cancer. These volunteers then designed a play, centred around the experiences of a woman who was diagnosed with late-stage cervical cancer and three other women from the village who accepted self-collection and received appropriate treatment, based on advice through health volunteers. This play was piloted among both health providers (doctors, nurses, social workers) as well as in two tribal villages and received positive feedback and suggestions for improvement. Based on the volunteers’ suggestions, this play was converted to drawings which will be used in the form of flipcharts/posters to motivate women to screen, after further feedback from stakeholders (figure in online supplemental additional file 2). A video explaining self-collection has been developed, while pictorial instructions on how to self-collect samples have been adapted with permission from the National Cervical Screening Programme, Australia.^32^

The messages will be delivered through group sessions, social media networks and local health facilities. Local champions at various levels (health providers, local leaders, local women and men) will be approached to promote cervical screening, to instil confidence and dispel myths. Based on community feedback, group-based education has been chosen as the main approach for education, utilising self-help groups, work site camps or special women’s meetings.

#### Screening management and data management

We will use canSCREEN, a screening registry software solution developed by the Australian Centre for the Prevention of Cervical Cancer, to record and manage participants’ episodes of screening, follow-up assessments and treatment of precancer.^33 34^ canSCREEN will be configured to meet the needs of the trial. The software facilitates standardised high quality data entry through the use of prespecified and study-specific drop-down menus (in language as required), is able to integrate laboratory results, produces reports and lists for action by result category, and can automate the management and sending of reminders and notifications by SMS. canSCREEN has been successfully used in projects in other low- and middle-income countries including Papua New Guinea, Malaysia, Kenya and Vanuatu.^33 35^

HPV negative results will be sent to the woman as an SMS or WhatsApp message (or other nominated preferred method if these options are unavailable or inappropriate), informing her of the need for repeat screening in 5–10 years. HPV positive women will be counselled at their homes and navigated to the next step by CHWs/volunteers using a set of structured messages and referral forms.

The trial data will be maintained on a secure server located in India, with restricted password protected access to designated investigators, to maintain confidentiality and data security, in accordance with Indian laws. Only the team at each site involved in follow-up and treatment of HPV positive women will be informed about the results. The final trial data will be under the custody of the Indian PIs, with only de-identified data made available for study analyses or later in a public data repository, with permission from the core study team.

#### HPV sample collection and NAT assays

Self-collected samples using dry swabs (Copan FLOQSwabs) will be transported periodically from each cluster to reach the laboratory at each site (Christian Medical College Vellore, Zoram Medical College, Aizawl, Tribal hospital, Sittilingi). HPV assays were chosen from the 20 currently clinically validated screening assays^36^ based on available equipment, feasibility of installation and testing at each site and utility for providing extended HPV genotyping. The 12 Group 1 carcinogenic HPV types have been recently grouped according to their prevalence in cervical cancer, which is a marker of their carcinogenicity, into Groups 1a (HPV 16: most carcinogenic), 1b (HPV types 18 and 45), 1c (HPV types 31,33,35,52,58) and 1d (39,51,56,59 - least carcinogenic).^37 38^ Currently, all clinically validated HPV assays also include HPV68 (a Group 2a – probably carcinogen) and/or HPV66 (a Group 2b - possible carcinogen). The results will be grouped based on genotyping, as shown in table 2 for the purpose of triaging for treatment. Alinity m HR HPV assay (Abbott Molecular, Abbott Park, USA) allows for extended genotyping to detect the seven Group 1a (HPV16), 1b (HPV18,45) 1c (HPV31/33/52/58) carcinogenic types that are included in the nonavalent HPV vaccine, while the Allplex HPV HR assay (Seegene, Seoul, South Korea) and Cepheid’s Xpert HPV test (Cepheid, Sunnyvale, USA) outputs allow for these seven HPV types as well as HPV35 (Group 1c) (table 2).^38 39^

**Table 2:** Details regarding laboratories and human papillomavirus (HPV) tests planned for the self-collected HPV evaluation for the prevention of cervical cancer trial.

Laboratory	Sites served	HPV assay, instrument	Classification of HPV positive results based on assay
Zoram Medical College, Aizawl, Mizoram	Mamit (tribal Mizoram)	Seegene Allplex, CFX96 Touch Real-Time PCR detection system (Bio-Rad)	Group 1a–c HPV types 16/18/45/31/33/52/58 ±35 Group 1d, 2a, 2b HPV types 39/51/56/59/66/68 ±35 (Group 1c)
CMC Vellore, Vellore, Tamil Nadu	Rural and urban Vellore; tribal Jawadhi hills	Alinity m HR HPV assay and system (Abbott)	
Tribal Health Initiative, Sittilingi, Tamil Nadu	Tribal Sittilingi valley	Xpert HPV assay and system (Cepheid)	HPV types identified per channel −16 (Group 1a)/18/45 (Group 1b)/31/33/35/52/58 (Group 1c); 39/51/56/59/66/68 (Group 1d, 2a, 2b)

HPV, human papillomavirus.

In the laboratory the dry swabs will be resuspended in MSwab media (#6E067N, 5 mL) for Seegene and Alinity m, and in 5 mL PreservCyt solution (Hologic, Marlborough, USA) for Xpert HPV testing, following standardised protocols.^40^ Samples eluted with MSwab media will be tested within 14 days if stored at room temperature, within 21 days if stored at 4°C and within 28 days at −20°C, with storage conditions depending on volumes of samples in each site. Specimens collected in PreservCyt Solution may be stored at 2–30°C for up to 3 months after the date of collection prior to performing the Xpert HPV test. Quality control in each laboratory will be undertaken by running known samples periodically to confirm accuracy.

#### Management of screen positive women

**Figure 3:**
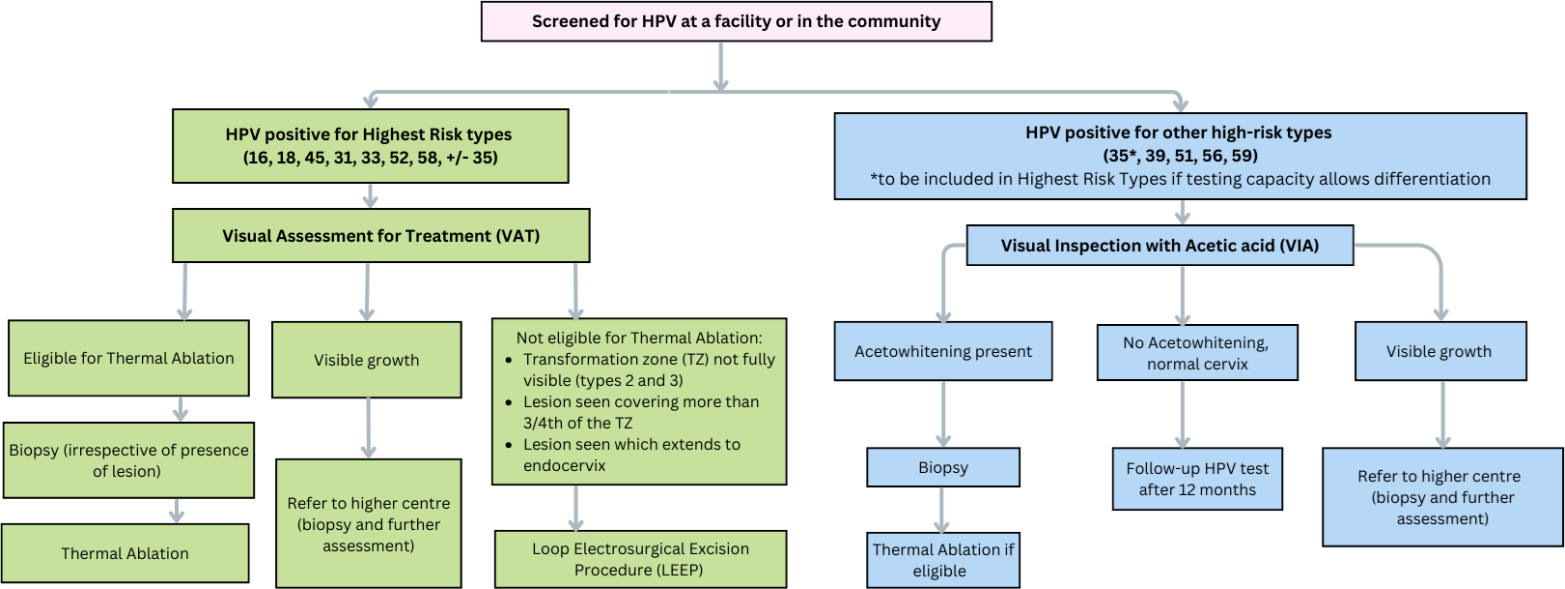
Protocol for management of HPV positive women. HPV, human papillomavirus.

Individuals who have any of the Group 1a-c HPV types detected (figure 3) will be offered visual assessment for treatment (VAT) at local health facilities (PHCs or district hospital in Mizoram, or the study organisations’ secondary hospitals in Tamil Nadu). VAT involves examination for eligibility for treatment using 3%–5% acetic acid application. Thermocoagulation will be used to treat screen positive women who are eligible, based on the following criteria:^30^

- The transformation zone (TZ) is fully visible, any lesion is wholly visible and not suspicious for cancer, and does not extend into the endocervix, or the lesion does not occupy more than 75% (3 quadrants) of the TZ.
- The woman has a type 1 TZ (entirely visible and only ectocervical), or
- The woman has a type 2 TZ (entirely visible and has an endocervical component), where the probe tip will achieve complete ablation of the SCJ epithelium, that is, where it can reach the upper limit of the TZ.

Those not eligible for thermal ablation will be referred to a gynaecologist for colposcopy, followed by biopsy or LEEP (loop electrosurgical excision procedure) as clinically indicated, at the same visit where possible. Cancer cases suspected at VAT or at colposcopy will be referred to local referral hospitals.

Those positive for less carcinogenic Group 1d HPV types (39, 51, 56, 59) ±35 will be offered triaging with VIA (figure 3). VIA positive women will be assessed for ablative treatment and offered the same, if eligible. VIA-positive women not eligible for ablation will be referred for colposcopy, followed by biopsy or LEEP as clinically indicated. VIA-negative women will be asked to return for a repeat HPV test in 12 months to assess clearance. Those with persistent Group 1d HPV (±HPV 35) infections will be offered colposcopy, followed by biopsy or LEEP as clinically indicated. Women with new positivity for Group 1a-c HPV types will be offered ablative treatment (following VAT), if eligible, or if ineligible, referred for further assessment at colposcopy.

Those who undergo any ablative treatment (VAT or post-VIA) will be offered a follow-up HPV test in 12 months.

#### Confirmation of underlying disease

All consenting HPV positive women requiring treatment will undergo biopsy before treatment, to confirm disease burden, positive predictive value of the tests and identify occult cancers. Biopsies will be transported in formalin to the pathology laboratory of the Christian Medical College Vellore or the Zoram Medical College. No biopsies will be taken from screen negative women, as determining sensitivity of the screening test is not an objective of this study, with these parameters well studied in multiple trials.

#### Training

Medical officers are being trained for management of screen positive women using thermal ablation, VAT and VIA triage, while gynaecologists are additionally being trained for colposcopy and LEEP. Other training includes training of nurses, social workers, community level workers, lab technicians, data entry operators and programme managers for various aspects such as community mobilisation, health education, counselling for follow-up, data management and laboratory processes.

#### Concomitant screening

Among those who undergo HPV screening at a facility, concomitant VIA, if performed based on the woman’s request, will be noted.

### Outcomes

The trial is a type 3 hybrid implementation research trial, which will primarily compare implementation outcomes while collecting data on clinical outcomes.^27^ Evaluation of the trial will use a mixed methods approach. The primary outcome will be the percentage of well-managed women, among all eligible women aged 30–49 years, measured 3 months from the last date of recruitment. Well-managed refers to having been screened using HPV testing and undergoing further assessment and treatment, based on results. Later analyses after the trial period will summarise long-term follow-up, including compliance with repeat HPV testing at 12 months. All quantitative implementation and effectiveness outcomes are outlined in tables3^5^.

**Table 3:** Quantitative outcome indicators at the individual level

Outcome	Indicator*	Formula for calculation
Primary outcome: Overall effectiveness of the programme	Percentage well managed (screened and managed appropriately) out of all eligible women in study area	Number of women who received appropriate assessment or treatment based on HPV results/total number of eligible women in study area
Screening coverage (reach)	Percentage of women screened with HPV testing among eligible women	Number of women screened with HPV testing/total number of eligible women in study area
Initial follow-up visit compliance	Percentage of HPV positive women who attend the first follow-up assessment	Number of HPV positive women who reported to a follow-up clinic/HPV positive women
Referral visit treatment compliance among those ineligible for ablation	Percentage of HPV positive women who attend a higher centre among those referred based on the initial follow-up assessment	Number of HPV positive women who underwent LEEP or appropriate management/HPV positive women who were referred to a higher centre based on ineligibility for ablation
Treatment rate (programme effectiveness in providing treatment)	Percentage of women treated among those HPV positive eligible for treatment	Number of HPV positive women who were treated/women who were eligible for treatment
12 month follow-up compliance	Percentage of women who complete repeat HPV testing at 12 months among women due for a 12 month follow-up HPV test	Number of women who complete a 12 month repeat HPV test/(number treated at baseline based on presence of highest risk genotypes or VIA positivity with other high risk genotypes+women with other high risk genotypes and negative VIA at baseline)
Acceptability	Percentage of screened women who would be willing to repeat self-collection or who would be willing to recommend the test to other women	Number who would be willing to repeat the test or would recommend the test/interviewed women who have completed screening
	Satisfaction rating for self-collection	Mean satisfaction score out of 5
	Percentage of women who underwent diagnostic or therapeutic procedures who experienced side effects leading to hospital visits	Number of screened positive women who reported side effects requiring hospital visits/number of screen positive women who underwent follow-up assessment

* Sources of information: screening registry for rates of screening and treatment; sample surveys for acceptability indicators.

HPV, human papillomavirus; LEEP, loop electrosurgical excision procedure; VIA, visual inspection of the cervix with acetic acid.

**Table 4:** Quantitative outcomes relating to health systems and settings

Outcome	Indicator	Formula for calculation	Means of verification
Adoption by providers and setting	Percentage of clusters that offered planned interventions	Number of clusters in which self-collection was offered to eligible women/clusters randomised to the specific arm	Programme records/study staff
Implementation fidelity for self-collection	Proportion of HPV testing that was done as self-collection	Number of women that performed self-collection/number of women screened using HPV testing	Screening registry
Implementation fidelity for approach to programme	Proportion of clusters in each arm that offered self-collection according to protocol	Number of clusters that offered screening in community or facility based on allocation/number of clusters in the arm	Programme records
Sustainability	Perceived sustainability of the programme approaches	The Intervention Scalability Assessment tool	Final stakeholder workshop

HPV, human papillomavirus.

**Table 5:** Additional quantitative process indicators

Process indicator	Indicator explanation	Formula or indicator for calculation	Means of verification
Invitation	Percentage of women aged 30–49 years who received information about screening among eligible women	Number of women aged 30–49 years who received information about HPV testing/total number of women aged 30–49 years	Sample survey at the end of the study period
Education session coverage	Percentage of clusters that offered at least one community-based educational programme and an appropriate number of small group sessions in each arm	Number of clusters that offered at least one community-based educational programme and an appropriate number of small group sessions (at least one session for every 50 women)/total study clusters per arm	Programme records
	Number of educational sessions organised in 12 months in each cluster	Mean number of sessions in each arm	
HPV sample adequacy	Adequacy of DNA samples received for testing	Number of inadequate samples received/total number of samples received	Screening registry
Implementation costs and cost effectiveness	Costs of invitation, obtaining samples, transport, laboratory testing, treatment, data management	Cost per woman screened and cost per HPV positive case treated	Micro-costing exercise

HPV, human papillomavirus.

We will also capture the quantity and costs of programme resources used in both arms, including costs of equipment, consumables, labour, transport, screening registry, educational materials and treatment of precancer. These costs will inform future cost-effectiveness analysis by comparing costs and effects of self-collection with VIA screening. Feedback surveys, including data on acceptability and possible harms, will be conducted among all consenting screen positive women and a random sample (10%, simple random sampling) of screen negative women from each site. Barriers and facilitators for providers and target women will be based on qualitative studies using the methodology described earlier.^22^ Focus group discussions (FGDs) with CHWs, key informant interviews with programme staff and interviews/FGDs with unscreened women and community members, including men, will be conducted to understand factors affecting implementation, challenges with screening and follow-up, as well as suggestions for future programmes. A final stakeholder workshop will be held to discuss trial findings and to assess sustainability using the Intervention Scalability Assessment tool.^41^

### Sample size and cluster randomisation

We assumed that around 40% would be well-managed in the facility arm, compared with 52% in the community arm, based on a previous study from India.^10^ A sample size of 2912 women (1456 per arm) to be recruited from a total of 28 clusters of mean cluster size 101 will provide 80% power to detect a 12% difference, assuming 5% alpha error, eight alpha adjustments, coefficient of variation between clusters of 0.69, and a design effect of 1.13 to account for variability in cluster size. The clusters will be distributed in four strata: 4 clusters from rural Tamil Nadu, 18 from tribal Tamil Nadu, 2 from urban Tamil Nadu and 4 from tribal Mizoram. The clusters in each of these four strata will first be grouped as blocks of 2, 4 or 8 clusters, according to proximity to the eight PHCs that are located in the study areas.

Block randomisation will be done to randomise clusters into Arm one and Arm two, using computer generated random numbers, by an offsite statistician, using Stata V.17.0, with allocation ratio as 1:1 in each arm. Although the trial will be an unblinded study, randomisation and revealing of the allocation sequence will occur only at the time of starting the trial. All eligible women in the study clusters will be invited to participate.

### Statistical analysis

The estimation of differences in the outcomes will be at the individual level (eg, percentage of women well managed, screened, treated, acceptability). Risk ratios obtained from modified Poisson regression models will be used to evaluate the effect of the intervention on the primary outcome (per cent well-managed). The risk ratio will be defined as r_2_/r_1_, where r_2_ will be the proportion of well managed in Arm one and r_1_ the proportion in Arm two. Adjustment for clustering will be included in both the univariate and multivariate regression models. We chose the modified Poisson regression model over the logistic regression approach due to its ability to provide estimates of relative risk rather than ORs.^42^

Analysis will be only at the end of the planned trial period, which is expected to last 8–12 months. The four subgroups that will be analysed are: rural Tamil Nadu, urban Tamil Nadu, tribal Tamil Nadu and Mizoram. Analysis of clusters will be by intention to treat. The trial will be reported using the Consolidated Standards of Reporting Trials checklist and its extension for cluster RCTs.^43^

### Study governance and community engagement

The project is governed by an Advisory Board consisting of senior representatives of the health departments of Tamil Nadu and Mizoram, senior clinicians, non-government representatives working for cervical cancer, and public health experts. A Steering Committee will actively monitor participation, HPV positivity rates, disease detection rates and any adverse events and troubleshoot and refine implementation in an ongoing way. As both arms use validated, standard clinical practices for screening and treatment, which are the same in both arms with only the location of screening differing, there is no standalone Safety and Data Monitoring Committee. This study differs from a formal intervention trial aiming at assessing a biological effect or efficacy outcome, in which the intervention is tightly prespecified and protocol deviations result in exclusion of participants. The implementation trial approach allows for deviations and adaption to maximise the likelihood of successful implementation of the intervention approach in the real-world setting. With careful documentation of fidelity to the intended implementation, implementation trials are able to generate key learnings about reasons for implementation success or failure and how and why any adaptations were necessary. We will document the nature of any modifications, rationale, timing and the decision-making process.^44^ Compliance with the trial protocol will be monitored, documented and managed by site specific study managers with oversight from Indian based CIs.

### Patient and public involvement

A community advisory group of around 4–8 members at each study site has and will continue to contribute to all study phases from co-design through to assessment of sustainability. Stakeholders include women residing in the study areas, tribal representatives, patient representatives, community leaders, local and state government health policy makers, health care workers and research community leaders.

### Ethics and dissemination

All study processes involve obtaining individual informed consent from participants as well as community consent from key community leaders. For this trial, each woman will receive only one round of screening. The informed consent process at the time of screening will include information about the research, its objectives and methods, information about the need for screening, the method used (self-collection), and options for treatment for those with a positive screening test, followed by written consent obtained by the CHW/research staff (form in online supplemental additional file 3). Separate consent will be obtained for treatment at the time of follow-up visit at the referral clinics, by the treating physician.

All aspects of the study will be conducted according to the amended Declaration of Helsinki. As a study funded by a foreign agency, we have obtained clearance from the Health Ministry Screening Committee (HMSC), under the Indian Council for Medical Research. Approval has been obtained from the district health authorities in both states.

Ethics approval has been obtained from the Institutional Review Board (IRB) and Ethics Committee of the Christian Medical College Vellore, Tamil Nadu, India (IRB Min. No 14314; INTERVEN), the Alfred Hospital Ethics Committee (HREC Ref 80134, Local Reference: project 601/21), Melbourne, Australia, the IARC Ethics Committee (IEC 21-32), Lyon, France, the Salem Polyclinic Institutional Ethics Committee (SPCIEC/2022/June/01/02), Tamil Nadu, India and the Institutional Ethics Committee, Civil Hospital, Aizawl, Mizoram, India (No.B.12018/1/13-CHA(A)/IEC/115). The study is also approved by the State Scientific Advisory Committee, Directorate of Public Health and Preventive Medicine, Chennai, Tamil Nadu (R. No. 011575/HEB/A2/2023). The Alfred Hospital Approval, as an authorised Australian ethics committee for national mutual recognition, is recognised and registered with the University of Melbourne Human Research Ethics Committee (2024-25255-57650-1).

The trial findings will be disseminated to the state governments, local health authorities, study communities, as well as to academic readers through the media, meetings, journals and conferences.

Recruitment of participants for the trial commenced on 21 March 2025 and is expected to be completed in all sites by June 2026.

## Discussion

Challenges to cancer screening in the Indian setting include a historical lack of a ‘culture of prevention’, inequality in healthcare access (especially for women), stigma and myths regarding cancer, all of which result in low uptake and follow-up, even when screening and treatment services are available.^4^

This trial is an implementation research trial that accepts a priori, on the basis of existing local and international evidence, that HPV-based screening is more effective than VIA screening^6^ and that self-collection is more acceptable than provider collection.^9^ Hence, we aim to compare implementation outcomes between two different approaches to providing self-collection in a community-based programme, through a Type 3 hybrid implementation research trial.^27^ Given that the trial will compare complex interventions delivered within existing healthcare systems, it will be a pragmatic trial,^45^ with a view to informing policymakers on possible approaches for programme delivery, with the study being powered to compare the primary outcome across the four strata (Mizoram, rural, tribal and urban slums in Tamil Nadu).

We hypothesise that utilising self-collection for primary screening will engage more women in screening given its high acceptability, allowing available resources and systems to focus on the effective provision of diagnosis and treatment pathways for the <10% of women who are HPV positive, resulting in more women being well-managed and ultimately reducing the risk of cervical cancer in vulnerable population groups.

We also hypothesise that community-based self-collection, which is closer to homes, will result in higher coverage than facility-based provider screening as offered currently in the national programme. Previous studies from India have demonstrated that home-based approaches are feasible and effective, as shown in studies from Andhra Pradesh, Tamil Nadu and West Bengal.^10 46 47^ Home-based screening offers the advantage of screening in the privacy of homes and reduces the costs and time involved in travelling to a health centre, especially for asymptomatic women who have low motivation for such screening. Such home or community-based screening (screening offered in a non-healthcare setting through mailed kits, door-to-door approaches or community campaigns) has been evaluated in many countries (eg, Italy, Mexico, India, Uganda, Rwanda, etc) and is now part of screening policy and routine practice in many countries (eg, Australia, the Netherlands, New Zealand, Sweden, Denmark, Timor Leste and England). This has led to improved screening rates among unscreened women.^8 9 16 18 48^

However, a door-to-door community-based approach implies individual counselling and a personal decision of the woman to screen. Our co-design phase revealed that in these low-resource settings where the trial is to be conducted, peers often influence decisions, and discussion and informed decisions may be better made collectively. Hence, the stakeholders preferred a camp-based approach that offers the possibility for group education and is sufficiently close to homes, such that women can either collect samples at the campsite in a private room/toilet or at homes (if nearby).

However, it is also possible that facility-based screening, as currently offered in India, will be easier to deliver and preferred, if self-collection is the test used, with data collected in our formative phase suggesting that some women, especially older women and previously screened women, favoured facility-based screening.

Our study is powered to detect a difference between the two arms in either direction (favouring either community or facility-based screening). Another study in Kenya also compared community campaign-based self-collection to facility-based self-collection, which was also based on the standard screening approach in the country.^49^ Given that the standard of care of cervical screening in India occurs through referral of women aged 30 years and above, by CHWs, for VIA screening at facilities (PHCs or HWC-health subcentres), Arm two adopts this programme approach.

Both trial arms are designed to be implementable in vulnerable populations and follow the same diagnostic/treatment approaches for women who return a positive screening test, although there are differences between sites based on differences in the health systems between each setting. We have chosen a Screen, Triage and Treat option for all the study sites based on our co-design phase.^39 50^ Current options for triage tests include cytology, p16/Ki-67 dual-staining cytology, partial genotyping (prioritising the most carcinogenic Group 1a-b HPV types 16 and 18 for further assessment or treatment), VIA and colposcopy±biopsy.^30^ Extended genotyping (inclusion of the next most carcinogenic types - Group 1c) to determine whom to treat based on risk has also been shown to improve sensitivity for the detection of CIN 3+ and facilitate safe deferring of colposcopy if the HPV types are of lower carcinogenic potential (Group d).^51 52^ A recent analysis highlighted the need to identify the eight most carcinogenic types in HPV-based screening.^39 53^

This will be the first RCT in India that will compare implementation outcomes of two different programme approaches for delivering self-collection. The only RCT on cervical cancer screening from India compared provider collected HPV-based screening to cytology and VIA-based screening, demonstrating the greater effectiveness of a single round of HPV screening in reducing mortality.^54^ One study offered home-based self-collection following an initial round of facility-based screening, showing a greater response for home-based screening (58% vs 46%),^10^ whereas another study reported that during the pandemic period home-based self-collection enabled maintaining the same numbers of women reached for screening as in the pre-pandemic period when facility-based provider collection had been used.^55^

The other unique aspect of our trial will be the focus on tribal women from the northeastern region, as well as from Tamil Nadu, a relatively economically advanced state with comparatively better-organised screening compared with most other states due to a long history of support from the World Bank. The proportion of tribal women in our study is larger than that of the other groups (22 out of 28 clusters) as we chose to focus on this vulnerable group, due to the lack of evidence regarding effective implementation of HPV-based cervical screening programmes in resource-poor settings, and among these vulnerable population groups. Tribal areas in both states have common challenges such as poor roads, high cost and distance of transport to secondary care facilities, representing areas that are least included in most studies.^56^

The trial is also poised to test a public-private partnership model for health promotion in Tamil Nadu, in an attempt to improve coverage and overcome difficulties in integrating a new screening approach into existing primary care services without overburdening existing health workers.

We acknowledge that our focus on low resource settings, and purposeful and restricted selection of eligible clusters based on operational considerations, will reduce the external validity of the findings. However, although the findings will not be generalisable to all women and settings across India, the trial will provide valuable lessons to policy makers in these and similar states regarding implementation outcomes of tailored approaches to future self-collection programmes.

## Supplementary material

10.1136/bmjopen-2025-101599 online supplemental file 1

10.1136/bmjopen-2025-101599 online supplemental file 2

10.1136/bmjopen-2025-101599 online supplemental file 3

## Data Availability

Data sharing not applicable as no datasets generated and/or analysed for this study.
