# Supplementary material for: Protocol for a cervical screening implementation trial comparing two approaches for delivering HPV self-collection in low-resource settings in India: a type 3 hybrid cluster randomised controlled trial (SHE-CAN)
Source: BMJ Open. 2025 Dec 29;15(12):e101599. doi: 10.1136/bmjopen-2025-101599 (PMC12750757; doi:10.1136/bmjopen-2025-101599)

Additional file 2 Fig. Storyline of co-designed educational material for participants

Part A. A woman who went for testing only when she had symptoms had to face the challenges of cancer treatment

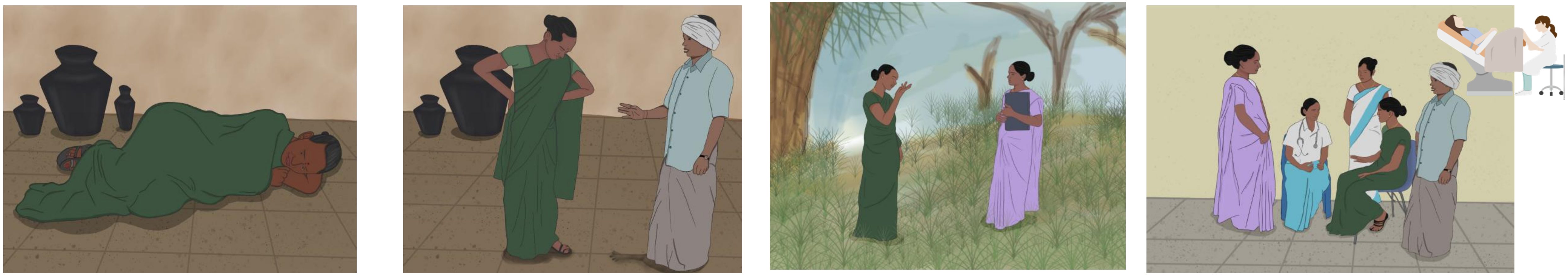

Part B. Women who decided to screen even without symptoms using self-collection were saved from cervical cancer. Health workers ensure women receive appropriate care based on HPV results obtained from the laboratory

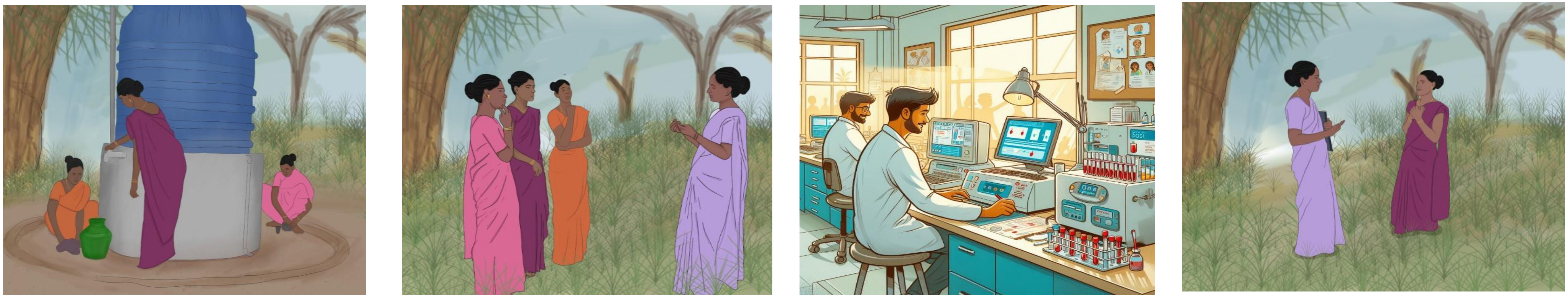

Supplement: online supplemental file 2 [file bmjopen-15-12-s002.pdf]
