# Supplementary material for: Protocol for a cervical screening implementation trial comparing two approaches for delivering HPV self-collection in low-resource settings in India: a type 3 hybrid cluster randomised controlled trial (SHE-CAN)
Source: BMJ Open. 2025 Dec 29;15(12):e101599. doi: 10.1136/bmjopen-2025-101599 (PMC12750757; doi:10.1136/bmjopen-2025-101599)
Supplement: online supplemental file 3 [file bmjopen-15-12-s003.docx]

**COMMUNITY HEALTH DEPARTMENT, CHRISTIAN MEDICAL COLLEGE VELLORE**

**PART I: PARTICIPANT INFORMATION SHEET - TRIAL**

**Study Title (lay title):**

Finding ways to improve cervical cancer screening programs for women using HPV testing (SHE-CAN study).

**Date: __/__/______**

**Introduction**

I am _____________________________________________, working for the study entitled “Finding ways to improve cervical cancer screening programs for women using HPV testing (SHE-CAN study)”.

We are conducting research on how to prevent cervical cancer, which is very common in Indian women. I am going to give you information and invite you to be part of this research. Your participation is entirely voluntary, and you are free to decide whether or not you would want to participate in the research. Before you decide, you can talk to anyone you feel comfortable with, including the research team about participation.

If there are some words that you may not understand, please ask me and I will take time to explain. If you have questions later also, you can ask me and the study doctor or the staff.

**Purpose of the research**

Cervical cancer refers to a growth in the cervix (the lower part of uterus), which leads to excess bleeding and white discharge, causing a lot of suffering in late stages and may cause death, if left untreated. Many women are diagnosed at late stages, when the disease is difficult to cure, and both the patient and the family suffer.

We would like to study the ways in which we can prevent women from getting this cancer or help women diagnose this condition early.

The best way to prevent or early diagnose the cancer is for women after 30 years of age to have a routine checkup called cervical screening. The cancer can be detected long before it develops by screening and can be treated. Our study will try to find the best community-based approach that can help many women who are living in villages or towns, where it is difficult to get good facilities for checkup and treatment.

The research study is being conducted under the guidance of Dr. Anu Mary Oommen, Professor, Community Health Department, Christian Medical College, Vellore, Tamil Nadu, India. The same study is also being conducted by Dr. Ravikumar Manoharan at the Tribal Health Initiative, Sittilingi and Zoram Medical College and Regional Cancer Center, Mizoram. The study is being supervised by Prof Julia Brotherton, The University of Melbourne, Australia and Dr. Partha Basu, Head, Early Detection, Prevention & Infection Branch, International Agency for Research on Cancer, World Health Organization, Lyon, France.

**Design of the study**

*Phase 1*

In the first year, we interviewed some women and other members of the community, to understand experiences and commonly faced barriers to accessing screening and treatment, preferences for screening, and to identify groups at high risk of not surviving after a diagnosis of cervical cancer. We also interviewed health care providers and visited health facilities, to understand the challenges in providing screening services.

*Phase 2*

We used all the information gained in Phase 1, to decide, along with the community and health providers, the options of how to practically provide cervical cancer screening in your locality using the currently recommended screening test – HPV test.

*Phase 3 (Trial phase, current study)*

We will divide the villages/urban wards into two groups for a trial using a chance process (similar to tossing a coin), and will offer the same HPV testing, to women aged 30-49 years in both groups. One group will be offered testing in their communities, while the other group will be offered testing at a health facility. We will see which approach helps more women get screened and receive appropriate treatment.

**Information about the intervention (screening test to prevent cervical cancer)/What you will be offered**

Women between 30 and 49 years of age residing in the villages/town areas that have been selected for the study, will be approached, and will get health education regarding the test. This test, also known as the Human Papilloma Virus (HPV) test is simple and very accurate. The test will let us know whether the woman has been infected by this HPV virus, which is the cause for cervical cancer. Although the virus infection is very common among married women and men, most of them naturally become free of this infection within a few months and years. For some women the virus remains in the body for many years and can eventually cause cervical cancer. A person who is HPV test positive has a higher chance of getting cervical cancer than a woman who is negative. However, not all women who are test positive will get cancer.

Sample collection for the test for detecting HPV infection requires inserting a swab stick with a soft bud (just like one used to take samples from the throat) into the vagina, either by the woman herself or a health worker, and taking some fluid for testing. All the women who consent to testing will be provided the test kit free of cost and will be taught how to use the swab.

If any woman tests positive, for most types of the virus, she will be offered treatment which involves internal examination of the cervix to see if she is eligible for free, simple outpatient treatment which takes a few minutes and can be done at the local health facility in your area. Women will also be offered a biopsy (removal of a small piece of the cervical tissue) before the treatment, to confirm whether there are any changes in the cells (pre-cancer). Treatment removes these cells, which stops cancer from developing. In case the internal examination shows that this simple treatment is not possible, you will be referred to a specialist for appropriate treatment. These are all the usual procedures already being followed by doctors when someone might have precancer or early cervical cancer. For some weaker types of the virus, the doctor will look at the cervix to make sure it looks healthy and if so, a follow-up test for the virus will be done in a year’s time to check the body has cleared the virus.

We would also like to ask you a few questions after the tests and also after the results, to understand your experiences, challenges faced and suggestions, through an interview either at home or in the clinic.

**Voluntary Participation**

Your participation in this research is entirely voluntary. It is your choice whether to participate or not. Whether you choose to participate or not, all the services you receive at home or clinic will continue and nothing will change. You may refuse participating in this trial even if you had agreed earlier and at any stage (like screening/diagnosis/treatment/follow up).

**Duration and sites**

The whole project will take place over three years in both rural, urban and tribal areas in Tamil Nadu and Mizoram.

**Risks**

There are no known risks with testing for HPV using the vaginal sampling kits other than very mild discomfort. If you test positive and need treatment, we will offer to treat you with standard diagnostic and treatment procedures followed in India and our hospital. Your doctor will explain any risks of this treatment should this be needed.

**Benefits**

If you participate in the screening study, you will know whether you have HPV infection and are at risk for future cervical cancer. If you are negative this will give you peace of mind. If you are positive this will help you decide whether you would like treatment for this infection which could prevent you from getting cancer in the future. Your participation will also help us work out the best way to provide screening to women like you which can help inform future services in your community and in other similar communities in India, which could save many women’s lives.

**Reimbursements**

No reimbursement for study participation in the research will be provided. If you need travel assistance for confirmatory tests and treatment, we will make the necessary arrangements (e.g. bus fare).

**Confidentiality and Sharing Results**

The information that we collect from this research project will be kept confidential. We will not be sharing the identity of those participating in the research. We will not publish any reports with your name or contact details.

Information about participants will be accessible only to authorized researchers and staff involved in delivering results and treatment.

The knowledge that we get from this research will be shared with you through community meetings and also will be made available to the scientific community and public through publication of research findings.

**Whom to Contact**

If you have any questions, you may ask me now, or any time later by contacting:

1. Name: Dr. Anu Mary Oommen

Professor, Community Health Department

Christian Medical College, Vellore, Tamil Nadu, India.

Mobile No: 9486368796

2. Name: Dr. Anne George Cherian

Professor (Gynaecologist), Community Health Department

Christian Medical College, Vellore, Tamil Nadu, India.

Mobile No: 9487760904

3. Name: Dr. Ruby Angeline Pricilla,

Professor, LCECU hospital,

Christian Medical College, Vellore, Tamil Nadu, India.

Mobile No: 9843128432

4. Name: Ms. Harini, SHE-CAN Project Manager

SHE-CAN Helpline number: 9585984739

For any feedback or concerns regarding this research, please contact the CMC IRB at 9092284294 (or) email to [irb@cmcvellore.ac.in](mailto:irb@cmcvellore.ac.in)

**COMMUNITY HEALTH DEPARTMENT, CHRISTIAN MEDICAL COLLEGE VELLORE**

**PART II: Consent form - TRIAL**

**Study Title:**

Finding ways to improve cervical cancer screening programs for women using HPV testing (SHE-CAN study).

**Study Number : ___________________________________**

**Participant’s Name :** ____________________________________

**Address :** ____________________________________

(Please tick boxes)

- I declare that I have read the information sheet provided to me regarding this study and have clarified any doubts that I had. [ ]
- I understand that the cervical screening tests in the study will be done free of cost and involves testing for the HPV virus using a sample of fluid from the vagina. I understand that if the test is positive, I will be advised regarding further testing or treatment, as needed, and helped to access these services. I also give consent to the study investigators for future follow up after the study ends, to enquire about my health.
- I also understand that my participation in this study is entirely voluntary and that I am free to withdraw permission to continue, to participate at any time without affecting my usual treatment or my legal rights. [ ]
- I understand that the study staff and institutional ethics committee members will not need my permission to look at my health records even if I withdraw from the trial. I agree to this access. [ ]
- I understand that my identity will not be revealed in any information released to third parties or published. [ ]
- I understand that the data collected in this study may be used for future related research, as long as that research has appropriate ethical approval, and I understand that my data collected as part of this study may be included, but without including any identifying information about me.
- I voluntarily agree to take part in this study. [ ]

**Participant:**

Name:

Signature Or thumb impression:

Date:

**Witness: Researcher:**

Name: Name:

Signature Or thumb impression: Signature:

Date: Date:

**PLACE:**
