# Supplementary material for: Protocol for a cervical screening implementation trial comparing two approaches for delivering HPV self-collection in low-resource settings in India: a type 3 hybrid cluster randomised controlled trial (SHE-CAN)
Source: BMJ Open. 2025 Dec 29;15(12):e101599. doi: 10.1136/bmjopen-2025-101599 (PMC12750757; doi:10.1136/bmjopen-2025-101599)
Supplement: Supplementary file 4 [file bmjopen-2025-101599.reviewer_comments.pdf]

PEER REVIEW HISTORY

BMJ Open publishes all reviews undertaken for accepted manuscripts. Reviewers are asked to complete a checklist review form and are provided with free text boxes to elaborate on their assessment. These free text comments are reproduced below.

ARTICLE DETAILS

Title (Provisional)

Protocol for a cervical screening implementation trial comparing two approaches for delivering HPV self-collection in low resource settings in India: a type 3 hybrid cluster randomized controlled trial (SHE-CAN)

Authors

Oommen, Anu Mary; Ashfaq, Maleeha; Tonsing, Mary Vanlalhruaii; Cherian, Anne George; Singarayar, Pravin; Viswanathan, Vidhya; Muniswamy, Venugopal; Hawkes, David; Abraham, Priya; Pricilla, Ruby Angeline; Manoharan, Ravikumar; Zomawia, Eric; Oldenburg, Brian; Saville, Marion; Krishnaraj, K; T S , Selvavinayagam; Basu, Partha; Brotherton, Julia ML; Collaborators, SHE-CAN

VERSION 1 - REVIEW

**Reviewer**	**1**
**Name**	**Ren, Wenhui**
**Affiliation**	**National Cancer Center/National Clinical Research Center for Cancer/Cancer Hospital, Chinese Academy of Medical Sciences and Peking Union Medical College**
**Date**	**11-Jun-2025**
**COI**	**None**

This protocol outlines a robust and novel cluster randomized controlled trial (RCT) design intended to address significant deficiencies in the implementation of cervical cancer screening, particularly within resource-limited settings in India. The study primarily concentrates on evaluating programmatic strategies for human papillomavirus (HPV) self-collection (community-based screening) in comparison to facility-based screening (comparator arm). Its objective is to furnish high-quality evidence regarding the implementation effects of HPV self-sampling within a community context. Nonetheless, certain aspects necessitate enhancement to ensure the research's quality.

1. Randomization process

Although the study is described as a cluster randomized controlled trial (RCT), the final 28 clusters were purposively selected from 83 eligible clusters based on their "feasibility for a

12-month trial." This approach may introduce selection bias and compromise the fundamental RCT principle of random sampling. Furthermore, the details of the randomization process have not been clearly articulated.

2. Sample Size Calculation

According to the established protocol, the determination of the sample size for each cluster, consisting of 200 women per cluster, is primarily influenced by operational considerations. It is recommended that statistical parameters also be taken into account in this process.

3. Statistical Analysis

The rationale for researchers selecting Poisson regression to analyze the percentage differences in effective management between arms. It is recommended to concurrently employ logistic regression analysis while taking into account the impact of central differences on the research outcomes.

**Reviewer**	**2**
**Name**	**Harper, Diane**
**Affiliation**	**Dartmouth Medical School**
**Date**	**24-Oct-2025**
**COI**	**None**

Comparative effectiveness research usually uses two approved strategies. In your protocol, you are using a home-based self-sampling strategy that does not have national approvals (FDA, EMEA, etc.). This protocol seems premature.

It is a well written protocol, but the fact that home based is not an approved method is worrisome. You cannot use this paper to state that home based is the same as clinic based collection.

The papers by Harper et have looked at home based vs. speculum based in the US, which you might be able to use to justify the home based arm.

VERSION 1 - AUTHOR RESPONSE

Reviewer 1	1. Randomization process<br>Although the study is described as a cluster randomized controlled trial (RCT), the final 28 clusters were purposively selected from 83	As the reviewer rightly pointed out, our process of selection of clusters has the potential to introduce bias due to the restricted process of selection of clusters. We have now acknowledged this as a limitation in the discussion.<br><br>Explanation:<br>We had to be pragmatic in selection of the clusters before randomising them into study and control arms as the study includes hard-to-reach rural and tribal areas with challenging	Page 30, last para

	eligible clusters based on their "feasibility for a 12-month trial." This approach may introduce selection bias and compromise the fundamental RCT principle of random sampling.<br><br>Furthermore, the details of the randomization process have not been clearly articulated.	geographical conditions. As both the CONSORT guidelines and the SPIRIT guidelines for protocols mention the need to report inclusion, exclusion criteria, as part of the process to assess eligibility (before the core step of randomisation), we have described the process of assessing eligibility.<br><br>We first selected 28 clusters from 83 eligible clusters depending on practicalities of conducting a trial, such as feasibility, staff availability, road access in tribal areas, and scattering of the population. After pre-selection of 28 study clusters based on operational feasibility, we randomly assigned them into 'study' and 'control' arms in 1:1 ratio.<br><br>We will also acknowledge the bias due to restricted selection of clusters for inclusion and the limited generalisability or external validity arising out of exclusion of the most hard-to-reach and geographically inaccessible clusters in our manuscripts to be published after completion of the study.<br><br>The randomisation process is described in page 24, para 1 as block randomisation, with the help of an external statistician using software. We have edited the para to give more details.	Page 10, para 2<br><br>Page 24 para 1
	According to the established protocol, the determination of the sample size for each cluster, consisting of 200 women per cluster, is primarily influenced by operational considerations. It is recommended that statistical parameters also be taken into account in this process.	We apologise for the confusion that this sentence caused. We have removed that sentence and reworded the para, as that was not the basis for sample size calculation. The statistical considerations for sample size are described later in page 23, and has been reworded for clarity.	Page 23
	Statistical analysis The rationale for researchers selecting Poisson regression to analyze the percentage differences in effective management between arms. It is	Thank you for the suggestion and we agree that we had not explained the rationale.<br><br>We would like to retain the modified Poisson regression as it will help to analyse the differences in terms of *relative risk* for clustered prospective studies like ours, while logistic regression will only give differences in terms of odds ratios.<br>We have added the rationale with the reference, to make it clear. Performance of the modified Poisson regression	

	recommended to concurrently employ logistic regression analysis while taking into account the impact of central differences on the research outcomes.	approach for estimating relative risks from clustered prospective data - PubMed	
Reviewer 2	Comparative effectiveness research usually uses two approved strategies. In your protocol, you are using a home-based self-sampling strategy that does not have national approvals (FDA, EMEA, etc.). This protocol seems premature.<br><br>It is a well written protocol, but the fact that home based is not an approved method is worrisome. You cannot use this paper to state that home based is the same as clinic based collection.<br><br>The papers by Harper et have looked at home based vs. speculum based in the US, which you might be able to use to justify the home based arm.	We thank the reviewer for identifying their concerns related to home-based self-collection and regulatory approvals. We agree that the trial cannot be used to compare self-collection with clinician-collection.<br>Our objective is not to compare the accuracy of the two arms but to compare the coverage achieved for screening and treatment in the two arms.<br><br>We are aware that the US has different regulatory assessment for self-collection undertaken within a clinical setting compared to outside a clinical setting, known as 'home-based'. However, a number of countries have a different regulatory approach to the US. Organised, national HPV-based cervical screening programs utilise 'home-based' (collection in a non-clinical setting) self-collection including Australia, The Netherlands, Sweden, Denmark, New Zealand, the Cook Islands, Nauru, TUvalua, Timor L'este and England.<br><br>https://pubmed.ncbi.nlm.nih.gov/34861338/<br><br>HPV-based cervical screening is only currently undertaken as part of research studies in India and only requires approval from scientific and ethics committees. Home-based self-collection for HPV-based cervical screening has been used in at least 9 studies in India (Strategies and implementation outcomes of HPV-based cervical screening studies to prevent cervical cancer in India: A systematic review - ScienceDirect) so far, including in a study by authors Oommen and Cherian (Strategies for primary HPV test-based cervical cancer screening programme in resource-limited settings in India: Results from a quasi-experimental pragmatic implementation trial - PubMed<br><br>As suggested by the reviewer, we have added references and a paragraph to explain that home or community-based screening has been tried in various countries and is an accepted part of organised routine screening programs in some, with references of reviews and studies from India, to justify the community-based arm.<br><br>Additionally, co-authors Hawkes and Saville were involved in the first self-collection methodology approved for home-based use in the US (Teal Health Wand) and are authors on the clinical	Page 28, first para

		validation paper Clinical Validation of a Vaginal Cervical Cancer Screening Self-Collection Method for At-Home Use: A Nonrandomized Clinical Trial - PubMed.<br>FDA Approves At-Home Self-Collection Device for Cervical Cancer Screening - The ASCO Post<br><br>Co-authors Hawkes, Brotherton and Saville are also authors of a self-collection validation study (SCoPE, Saville et al, JCV, 2020 https://pubmed.ncbi.nlm.nih.gov/32361328/) which is cited in the BD Onclarity HPV test Instructions for Use, with BD submitting an application to the FDA for home-based self-collection in the US in 2025.<br><br>Thus, we would like to reassure the reviewer that the community-based approach that is being tried in this study has already been used in multiple other countries including India. Although the results may not be applicable to the US, they will have value to other countries, especially LMICs where unscreened women hesitate in visiting facilities when asymptomatic.<br><br>The methods used in the SHE-CAN study (abbreviated to the HPV assays - Alinity m, Xpert and Seegene) have been validated by the Australian HPV Reference Laboratory led by investigators Hawkes and Saville. The Xpert self-collection has been published (Saville et al,, JCV, 2020 https://pubmed.ncbi.nlm.nih.gov/32361328/) and the Alinity m and Seegene clinical validations (SCoPE2 study) have been presented at international conferences (EUROGIN, 2024 and IPVC, 2024) with manuscripts under preparation.	
